# Combined Therapy of Posttraumatic Stress Disorder with Stellate Ganglion Block and Radiofrequency Ablation in Military Patients

**DOI:** 10.1192/j.eurpsy.2026.11875

**Published:** 2026-07-31

**Authors:** S. Moroz, V. Semenikhina

**Affiliations:** Municipal Enterprise Dnipropetrovsk Multiprofile Clinical Hospital For Provision Of Psychiatric Care » Dnipropetrovsk Regional Council, Dnipro, Ukraine

## Abstract

**Introduction:**

Post-traumatic stress disorder (PTSD) is one of the most prevalent and disabling mental health consequences among military personnel exposed to combat. Traditional therapeutic strategies, including psychotherapy and pharmacotherapy, often provide delayed or incomplete relief, with up to 40% of patients remaining symptomatic. Novel interventional approaches such as stellate ganglion block (SGB) and radiofrequency ablation (RFA) of the stellate ganglion have shown promise in reducing sympathetic hyperactivity and alleviating PTSD symptoms.

**Objectives:**

This pilot study aimed to evaluate the clinical efficacy and safety of combined therapy using stellate ganglion block and radiofrequency ablation as an adjunct to standard multimodal care in military patients with severe PTSD.

**Methods:**

From May to July 2025, 61 male military patients (mean age 42.4±9.8 years) with clinically confirmed PTSD (Mississippi Scale score >100) were enrolled. Exclusion criteria included structural brain lesions on CT/MRI. Twenty-four patients consented to interventional treatment (SGB and/or RFA) in addition to standard therapy (psychotherapy, supportive physiotherapy, and nutrition). Thirty-seven patients served as controls, receiving only standard treatment. Procedures were performed under fluoroscopic guidance with intraoperative stimulation for accurate localization. Clinical outcomes were assessed using the Mississippi PTSD Scale and quantitative EEG (dominant alpha/beta rhythm ratio) before intervention and at day 14. Safety outcomes included intra- and postoperative complications.

**Results:**

In the intervention group, PTSD severity decreased by 41 points on the Mississippi Scale compared to 22 points in controls (p<0.001). EEG analysis revealed a 41% restoration of alpha rhythm in the intervention group versus 9% in controls (p<0.001), with a parallel reduction in beta rhythm (−38% vs −13%). Vegetative symptoms (hyperhidrosis, blood pressure fluctuations) markedly improved in the intervention group by days 14–21. Clinical response rate to SGB/RFA reached 94.6%. The most frequent expected side effect was transient Horner’s syndrome; one case of self-limited generalized seizure (4.1%) was reported.

**Conclusions:**

Stellate ganglion block and radiofrequency ablation represent effective and relatively safe adjunctive interventions for severe PTSD in military patients. These techniques offer rapid symptom reduction (1–2 days), sustained benefit (up to 3 weeks), and objective neurophysiological improvement, making them valuable options in multidisciplinary hospital settings. Further large-scale, controlled studies are warranted to confirm long-term efficacy and broaden applications to psychosomatic disorders.

**Disclosure of Interest:**

None Declared

